# WHAT STORIES DO FILMS TELL ABOUT AGING?

**DOI:** 10.1093/geroni/igz038.1515

**Published:** 2019-11-08

**Authors:** Helen Q Kivnick, Jim Vandenbosch

**Affiliations:** 1 University of Minnesota School of Social Work, Saint Paul, Minnesota, United States; 2 Terra Nova Films, Chicago, Illinois, United States

## Abstract

With the aging of the worldwide population, films inevitably include an increasing number of stories about aging and its vicissitudes. Gerontologists acknowledge that aging (a process that is at once biological, social, and psychological) includes elements of decline, loss, and fear– along with opportunities for new learning and creativity, contributions, love, and satisfactions. However given mainstream society’s predominant demand for the status quo, we should not be surprised that films made for popular, commercial audiences tend to reflect self-reinforcing, negative stereotypes and defenses against them (e.g., denial; fantasy; melodrama). This symposium seeks to: 1) Name and illustrate this filmic process of reinforcing negative stereotypes and misconceptions; 2) Describe and illustrate two existing theories that. explain positive processes associated with healthy aging; 3) Identify and illustrate films that constitute positive alternatives to the status quo and, in so doing, open the door to films presenting realistic stories of the positive balancing of later-life Integrity with Despair. The first presenter will articulate and illustrate this filmic process of reinforcing negative stereotypes and misconceptions. Presenters #2 and 3 will describe and illustrate two existing theories that explain positive processes associated with healthy aging (Baltes & Baltes’ Selective Optimization and Compensation; Erikson’s Integrity & Despair). Presenter #1 will return to identify and illustrate films that constitute positive alternatives to the status quo and, in so doing, open the door to discussing films that present realistic stories of older adulthood’s complexities.

